# EMPOWERING OLDER ADULTS: A PARTICIPANT’S PERSPECTIVE ON VIVO’S ONLINE STRENGTH TRAINING PROGRAM

**DOI:** 10.1093/geroni/igae098.1033

**Published:** 2024-12-31

**Authors:** Jennifer Pettis

**Affiliations:** Gerontological Society of America, Clifton Park, New York, United States

## Abstract

This abstract presents the narrative of a participant, detailing her positive experiences with Vivo, an online live and interactive strength training program. This presentation will illuminate the transformative impact of Vivo’s holistic approach to fitness among older adults. The participant, enthusiastically recounts her journey with Vivo, highlighting remarkable gains in muscle strength and energy levels. Engaging in Vivo’s tailored workouts, she experienced tangible improvements in her physical abilities, allowing her to navigate daily tasks with newfound ease and confidence. Notably, she emphasizes the program’s adaptability to her age group, catering to varying fitness levels while promoting safe and effective exercise practices. Beyond physical benefits, the participant underscores the significance of socialization within the Vivo community. Participating in live sessions facilitated meaningful connections with peers, fostering a supportive network that transcended geographical boundaries. These interactions not only enhanced her motivation but also nurtured a sense of belonging and camaraderie, vital components of holistic well-being among older adults. In conclusion, the participant’s narrative exemplifies the transformative power of Vivo’s online strength training program for older adults, showcasing its capacity to enhance muscle strength, energy levels, and social connectedness. This perspective underscores the program’s potential to promote active aging and improve overall quality of life among older adult populations.

